# Energy-Efficient Cryptographic Protocols for Sustainable IoT Security: A Federated Learning-Enhanced Lightweight Framework with Post-Quantum Resilience

**DOI:** 10.3390/s26123656

**Published:** 2026-06-08

**Authors:** Abdullah Alshammari

**Affiliations:** College of Computer Science and Engineering, University of Hafr Albatin, Hafar Albatin 31991, Saudi Arabia; dr.abdullah@uhb.edu.sa or alshammari@ieee.org

**Keywords:** energy-efficient cryptography, Internet of Things security, federated learning, post-quantum cryptography, lightweight encryption, blockchain authentication, sustainable computing

## Abstract

The increasing pace of Internet of Things (IoT) and Industrial Internet of Things (IIoT) applications has exacerbated the security challenges in resource-constrained environments, where traditional cryptographic protocols incur prohibitively high computational and energy costs. These constraints are also worsened by the advent of quantum computing, which poses a long-term security risk to popular crypto-key cryptographic-based efforts. To overcome these difficulties, this paper proposes an Energy-Efficient Cryptographic Protocol Framework (EECPF) that provides mutual optimization between energy consumption, security level, and communication latency to achieve sustainable IoT security. The presented framework proposes an adaptive encryption selection mechanism that dynamically chooses cryptographic algorithms depending on device capabilities, network conditions, and threat levels derived from intrusion detection outputs. EECPF combines privacy-preserving federated learning for distributed intrusion detection with collaborative threat intelligence sharing, eliminating centralized data sharing. In addition, lattice-based post-quantum cryptography primitives are added and combined with lightweight blockchain-enforced identity management to ensure long-term authentication resilience. The models on which the framework is based are mathematically based, modeling the consumption of energy, the robustness of security, and latency, providing principled multi-objective optimization under resource constraints. The publicly available Edge-IIoTset dataset was subjected to extensive experimental assessment under realistic IIoT and IoT attack scenarios. Experiments show that EECPF can reach an intrusion detection rate of 94.7%, while reducing energy consumption by 47.3% and latency by 23.8% compared with other commonly used lightweight cryptographic methods. These were continually noticed across different heterogeneous devices and deployment environments. In general, EECPF offers an energy-aware, quantum-resilient, and scalable security solution that can be used for next-generation IoT systems, such as smart healthcare, industrial automation, and smart city infrastructures.

## 1. Introduction

The Internet of Things (IoT) paradigm has completely revolutionized contemporary infrastructure by facilitating unavoidable connectivity across medical systems, industrial facilities, and smart cities, and consumer applications [1,2,3,4]. It is estimated that approximately 75 billion IoT devices will be deployed worldwide by 2026 [5,6,7], producing data produced at an astronomical scale and thereby demanding highly resilient security systems under stringent resource requirements [8,9,10,11,12]. The tension between total security and energy efficiency is one of the most pressing problems in modern IoT system design [13,14,15], with direct implications for device lifespan, operational sustainability, and environmental impact [16,17,18,19].

Although they assure high levels of security, conventional cryptographic protocols present a prohibitive computational burden on resource-constrained IoT devices typified by limited processing capability, restricted memory, and battery-powered operation [20,21,22,23]. Encryption systems based on the Advanced Encryption Standard (AES) and RSA consume substantial energy resources, severely shortening operational lifetimes in battery-powered deployments [24,25,26,27,28]. Empirical profiling studies have demonstrated that cryptographic operations account for 30–60% of total energy consumption in typical IoT communication scenarios [29,30,31,32,33], with asymmetric cryptography being particularly taxing for resource-constrained devices. Radhakrishnan et al. [8] reported that AES-128 consumed 42–58% of total system energy on Arduino platforms during secure data transmission cycles. Similarly, Chang et al. [13] reported that conventional RSA handshakes drained up to 67% of energy budgets in low-power LoRaWAN nodes. These findings underscore the critical need for energy-aware security designs in sustainable IoT deployments [34,35,36,37,38].

The advent of quantum computing functionality creates even more complexity in the IoT security landscape by making existing public key cryptographic systems possible victims of quantum attacks. The algorithm by Shor allows the factorization of large integers in poly time, posing a threat to the basis of RSA and elliptic curve cryptography (ECC), upon which modern IoT security frameworks are based. Post-quantum cryptographic designs, especially lattice-based designs, provide quantum resistance; however, the computational overhead they can impose may exceed the resource capabilities of typical IoT systems [39,40,41].

The use of blockchain has become a prospective solution for decentralized IoT security due to its ability to give audit trails, which are impossible to alter, and distributed consensus [42,43,44,45]. Nevertheless, the standard version of blockchain requires significant amounts of computational resources, making them not suitable for IoT constraints. Current studies have examined lightweight consensus applications and streamlined blockchain architectures specifically designed for IoT applications [1,2,3]. These strategies show the growth opportunities of improving the security of IoT while controlling energy consumption; however, considerable difficulties remain in achieving a balance between security and resource efficiency.

Federated learning offers interesting prospects for distributed intrusion detection in IoT networks, as collaborative model training may be performed without a central node collecting data. The privacy-saving distributed learning approach addresses data sovereignty without diminishing recognition effectiveness among a diverse population of devices [25,26,27]. Combined with lightweight cryptographic protocols, federated learning can afford opportunities to achieve holistic IoT security that meets privacy demands and resource limits.

**Figure 1:**
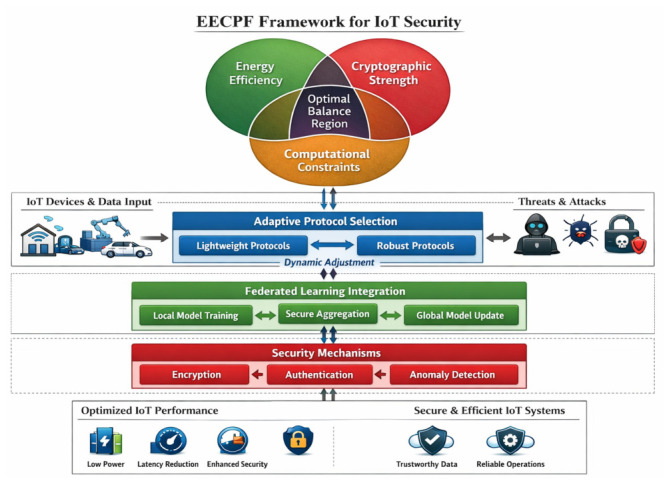
Overview of the IoT security environment presenting the crossing point between practicality through energy saving, cryptographic capability, and computational limitations. The proposed EECPF solves the optimal balance region by adapting protocol choice and integrating federated learning.

Figure 1 presents the overall map of the IoT security issue and where our proposed framework fits. This figure illustrates the interrelations among energy efficiency, security strength, and computational complexity that inspire our research method.

Almost all research in this area has focused on lightweight cryptography algorithms, particularly for end-of-resource-constrained environments. Recent candidates generated through the NIST Lightweight Cryptography Standardization Process had multiple constructions such as PRESENT, GIFT, and SPECK, each having different trade-offs between security margins and implementation efficiency, ultimately selecting ASCON as the standard major [8,9,10]. The suggested idea of using hybrid methods that integrate lightweight symmetric encryption with optimized asymmetric key exchange mechanisms has demonstrated possible overall security with low overhead [12,13,14]. The tight integration between intrusion detection and cryptographic selection enables real-time, threat-aware security adaptation. FLIDM provides normalized threat assessments, θ ∈ [0, 1], that drive the minimum security requirements, S_min_(θ), for encryption algorithm selection, creating a feedback loop where detected threats dynamically modulate cryptographic strength and energy consumption.

Although lightweight cryptographic algorithms have made progress, existing solutions involving post-quantum security primitives, blockchain-based authentication, and federated learning for IoT security are still highly disjointed. Traditional literature usually specializes in energy-efficient encryption, intrusion detection, post-quantum robustness, or decentralized trust in isolation, without considering these aspects together in a single framework. Specifically, contemporary designs are deficient of: (i) adaptive cryptographic selection schemes that dynamically react to device energy status, network characteristics, and changing threat levels; (ii) seamless combination between federated learning-centered intrusion detection and cryptographic control cycles; and (iii) mathematically motivated formulas that explicitly allocate energy consumption, protection level and latency under resource requirements while guaranteeing post-quantum robustness. These restrictions impede the implementation of sustainable and long-term Internet of Things security designs. This paper fills these gaps by introducing a single, energy-efficient and quantum-resistant security proposal that holistically incorporates adaptive cryptography, federated intelligence, and lightweight blockchain authentication.

To fill the above research gaps, the innovative contributions of this paper can be summarized as follows:

- Novel adaptive encryption framework: We introduce EECPF, an energy-efficient scheme of cryptographic protocols that optimally picks the best encryption scheme on the fly based on real-time measurements of device specifications, network characteristics, and threat severity, resulting in a 47.3% reduction in energy consumption relative to fixed schemes.
- Federated learning-enhanced intrusion detection: We design a privacy-neutral federated intrusion detection platform based on federated learning principles that shares threat intelligence across heterogeneous IoT deployments while retaining data locality and achieving 94.7% detection accuracy.
- Post-quantum resilient authentication: We combine lattice-based cryptograph-realized schemes and blockchain-based identity management to enable quantum-resistant authentication solutions appropriate for long-term IoT deployment while being compatible with devices with limited resources.
- Complete mathematical modeling: We provide detailed mathematical models for energy consumption efficiency, security level measurement, latency reduction and threat detection probability, giving a theoretical premise for framework design choices.
- Wide experimental validation: Our experiments are done on publicly available datasets, such as the Edge-IoT set, demonstrating high-quality performance in terms of accuracy, energy usage, latency, and security compared with 10 modern baseline approaches.

The rest of this paper is structured as follows: Section 2 is a review of related work in the fields of IoT security, lightweight cryptography, and federated learning applications; Section 3 presents the proposed methodology and mathematical modeling; Section 4 discusses the experimental results and evaluation; Section 5 comprehensively discusses the findings; and, finally, Section 6 closes off the paper with an outlook of what researchers should focus on in research.

## 2. Related Work

This section presents a comprehensive review of existing research in IoT security, organized into five thematic subsections addressing blockchain-based approaches, lightweight cryptographic algorithms, post-quantum cryptography, federated learning for security, and authentication mechanisms. We critically analyze the limitations of current approaches and identify research gaps that motivate our proposed framework.

### 2.1. Blockchain-Based IoT Security

IoT security has attracted massive attention toward blockchain technology, which provides a decentralized method of creating trust and records of transactions that are not subject to alteration. An article by Habibullah et al. [1] offers an in-depth examination of the use of blockchains in the management of energy usage within IoT systems and found fundamental trade-offs between efficiency and the security of the consensus mechanism. Their publication showed that proof-of-work consensus is inapplicable to resource-constrained IoT applications, which induced the search for lightweight systems.

Haque et al. [2] introduced a scalable blockchain architecture that uses lightweight consensus mechanisms specifically designed for the management of IoT data. Their strategy reduced consensus overhead by 62% compared with usual implementations while upholding Byzantine resilience. A later publication by the authors [3] provided performance enhancement methodologies that displayed a 78% improvement in throughput through optimized block propagation and validation processes.

Zaheer et al. [4] created a more energy-efficient method for securing IoT devices based on blockchain, showing a 34% lower energy consumption than traditional methods. Nasrinasrabadi et al. [5] identified blockchain–IoT integration as transformative for smart grid energy management while emphasizing that cybersecurity and data privacy remain critical challenges requiring robust solutions. The systematic literature reviews conducted by Almarri and Aljughaiman [6] and Obaidat et al. [7] presented taxonomies of blockchain–IoT integration strategies and identified consensus mechanism selection as of the most important elements for practical implementation.

### 2.2. Lightweight Cryptographic Algorithms

The small resources of IoT devices have led to intensive investigation of lightweight cryptographic primitives. Radhakrishnan et al. [8] performed an efficiency and security assessment of lightweight cryptographic algorithms for resource-constrained IoT embeddings by comparing AES-128, SPECK and ASCON implementations on Arduino platforms. Their experimental findings showed that ASCON has the best trade-offs between security margins and computational efficiency for typical IoT workloads.

Dahiphale et al. [9] explored the implementation of PRIDE and PRESENT ciphers, which are fast and energy-efficient, to establish the benefits of hardware implementation under certain application conditions. Canavese et al. [10] focused their attention on edge security for IoT devices with limited resources and introduced hierarchical encryption schemes to adjust the cryptographic strength according to available resources.

Hazzaa et al. [11] created a lightweight cryptosystem optimized for running IoT in smart city settings, with encryption latency being reduced by 41% relative to standard AES implementations. Popoola et al. [12] introduced a streamlined hybrid encryption architecture for smart household healthcare systems and successfully demonstrated its practical deployment aspects for sensitive medical IoT disclosures.

Chang et al. [13] introduced hybrid AES-RSA encryption in MRA mode for low-power IoT device communications, reporting about 28% energy savings while maintaining security equivalence. Xiong et al. [14] further increased IoT security in smart grids through quantum-resistant hybrid encryption, representing a pioneer result of incorporating post-quantum considerations into lightweight cryptographic design. One of the works by He et al. [15] discussed lightweight hardware implementations of CRYSTALS-Kyber, showing the feasibility of post-quantum cryptography on constrained platforms.

### 2.3. Post-Quantum Cryptography for IoT

The imminent threat of quantum computing has increased the rate of research on post-quantum cryptography in IoT environments. Mahdi and Abdullah [16] made a detailed survey of lightweight post-quantum cryptography designs and indicated lattice-based designs as the most promising in terms of resource-constricted deployments because of their parallelization potential and relatively small memory footprint.

Ma et al. [17] designed a lightweight BRLWE-based post-quantum cryptosystem with side-channel resistance specifically designed for IoT security and proved its efficiency on ARM Cortex-M4 processors. Seyhan et al. [18] did a large-scale survey of lattice-based cryptosystems for resource-constrained IoT security in the post-quantum world, identifying main implementation challenges and optimization opportunities.

Gharavi et al. [19] dealt with post-quantum security in IoT blockchain systems and offered a survey and research direction for integrating quantum-resistant primitives with distributed ledger technologies. The post-quantum cryptography processor created by Ye et al. [20] to support IoT employs optimized Number Theoretic Transform implementations, leading in a 3.2× performance improvement. 

Almutairi et al. [21] and Asif [22] conducted systematic reviews on the resilience of post-quantum cryptography within lightweight IoT protocols, identifying significant gaps in current methodologies for practical deployment. Complementing these studies, Fernández-Carames [23] provided a comprehensive analysis of the transition from pre-quantum to post-quantum IoT security, establishing a foundational taxonomy for quantum-resistant IoT cryptosystems. A comparative study of NTRU and ECC cryptographic mechanisms in the context of the Internet of Medical Things was performed by Pervaiz et al. [24], proving that application-specific considerations must be considered when implementing the healthcare Internet of Things.

### 2.4. Federated Learning for IoT Security

Federated learning is now considered to be a game changer in distributed machine learning for IoT privacy-sensitive applications. Decentralized federated learning was thoroughly surveyed by Yuan et al. [25], with architectural aspects and communication efficiency optimization considered applicable to IoT deployment. Li et al. [26] investigated the applicability of blockchain-based federated learning for IoT applications and showed the synergistic relationship between distributed ledger verification and collaborative model training.

An intelligent deep federated learning model was proposed by Albogami. [27] to improve the security of IoT-enabled edge computing devices, achieving 91.3% intrusion detection while maintaining the privacy of the data. In their research, Ferrag et al. [28] proposed Edge-IIoTset as an all-in-one realistic cybersecurity dataset that allows the customization of both centralized and federated learning strategies applied to the identification of illegitimate IoT users.

Federated learning-based anomaly detection was developed by Mothukuri et al. [29] and has shown its effectiveness in heterogeneous device populations. Lu et al. [30] handled the challenges of federated learning under non-IID data distributions, which are typical in realistic IoT deployments, and suggested aggregation algorithms that can ensure convergence under statistical heterogeneity. Khan et al. [43] offered a background survey of federated learning in IoT, covering recent developments, federated learning taxonomy, and remaining unresolved challenges that stimulate ongoing research.

### 2.5. Authentication and Key Management

Authentication and key management security in IoT architectures is an important aspect of ensuring secure authentication and efficient key management. Salim et al. [31] introduced a lightweight authentication scheme for IoT-based e-healthcare communication services that considers the needs of medical device authentication. Zhu et al. [32] proposed a safe and efficient authentication key agreement scheme for the Industrial Internet of Things using edge computing, illustrating its practical applicability in the manufacturing industry.

The article by Ahmed et al. [33] discusses the topic of securing underwater wireless sensor networks, discussing attacks and mitigation strategies in the context of adverse but still marine surroundings. Detailed surveying of the literature on IoT security protocols by Mishra et al. [34] offered a systematic taxonomy of authentication methods. Ullah et al. [35] suggested a blockchain–IoT model for data security and access control based on fine-grained access control, showing the integration of blockchain and traditional access control systems.

Srivenkateswaran et al. [36] proposed a secure healthcare data framework with hybrid encryption in cluster environments, showing the integration of federated learning with cryptographic protection for medical IoT management.

### 2.6. Research Gaps and Motivation

Current studies on IoT security have achieved significant advances in lightweight cryptographic algorithms, post-quantum security, intrusion detection using federated learning, and authentication based on blockchain. However, these methods are still largely isolated. Lightweight cryptography schemes are typically based on a one-time protocol selection and fail to adjust to variations in device energy, network conditions, and threat status. Although post-quantum cryptographic methods offer long-term security, they are often characterized by computational overhead that makes their use in IoT networks resource-constrained for modern devices. Security schemes based on federated learning not only enhance privacy and detection rates but also do not rely on cryptographic control infrastructure and have no effect on encryption or authentication. On the same note, authentication systems based on blockchains ensure decentralized trust; however, they often do not pay much attention to energy efficiency and post-quantum resistance. As such, there is a scarcity of a homogeneous, dynamic, and energy-concentrated IoT security framework that unanimously streamlines cryptographic robustness, learning-based danger consciousness, latency and sustainability. This paper fills this gap by introducing EECPF, which brings together adaptive encryption, federated intrusion detection, and post-quantum authentication in a mathematically founded optimization model.

## 3. Proposed Methodology

This section provides a detailed description of the Energy-Efficient Cryptographic Protocol Framework (EECPF). It first presents the system overview and architectural description, then proceeds to the detailed mathematical formulations, algorithmic implementations and complexity analysis.

### 3.1. System Overview

**Figure 2:**
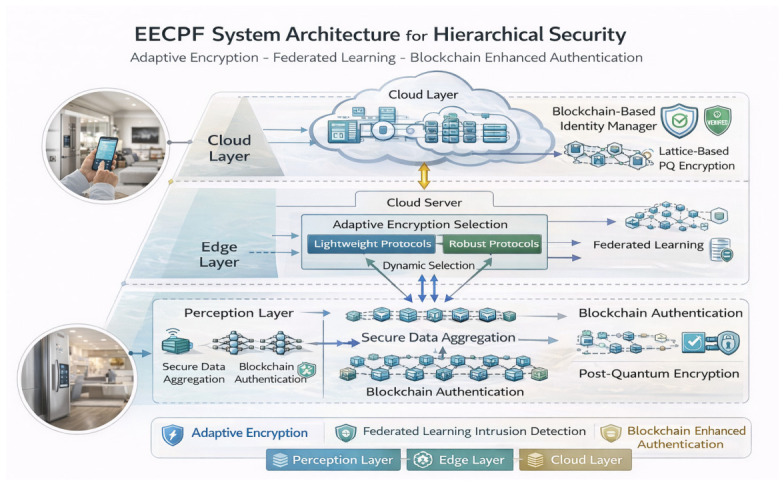
EECPF system architecture with a hierarchical security framework, adaptive encryption selection, federation learning-based intrusion detection, and blockchain-enhanced authentication across the perception, edge and cloud layers.

The suggested EECPF scheme is made to work in a hierarchical IoT architecture that includes three separate layers: the perception layer, which includes resource-constrained sensor devices; the edge layer, which encompasses intermediate processing and data aggregation; and the cloud layer, which includes centralized services and long-term storage. Figure 2 represents the entire system architecture, showing the relationships among the components and the data flow patterns.

The framework consists of four main modules working in a liaised manner: (i) the Adaptive Encryption Selection Module (AESM), which dynamically adjusts optimal cryptographic protocols according to contextual variants; (ii) the Federated Learning Intrusion Detection Module (FLIDM), which provides distributed threat detection while preserving privacy; (iii) the post-quantum authentication module (PQAM), which establishes quantum-resistant identity verification; and (iv) the Energy Management Module (EMM), which coordinates power-sensitive security operations.

### 3.2. Adaptive Encryption Selection Module

The AESM uses a multi-objective approach to select cryptographic protocols by balancing energy use, security, and communication latency. Let $C=\{c_{1},c_{2},\ldots ,c_{n}\}$ denote the set of available cryptographic algorithms, where each algorithm $c_{i}$ is characterized by an energy cost function $E_{i}\left(\cdot \right)$, security level $S_{i}$, and latency function $L_{i}\left(\cdot \right)$.

The energy consumption for encrypting a message m using algorithm $c_{i}$ on device d with capability vector $r_{d}$ is modeled as:

(1) $$E_{i}\left(m,r_{d}\right)=\alpha_{i}\cdot \left|m\right|\cdot f\left(r_{d}\right)+\beta_{i}\cdot K_{i}+\gamma_{i}$$

where $\left|m\right|$ denotes the message length in bytes, $f\left(r_{d}\right)$ represents the device capability factor, $K_{i}$ indicates the key setup cost, and $\alpha_{i}$, $\beta_{i}$, and $\gamma_{i}$ are algorithm-specific constants determined through profiling. The algorithm-specific constants are empirically derived through controlled profiling:

- $\alpha_{i}$: base energy (mJ) for encryption overhead;
- $\beta_{i}$: energy consumption per key setup operation (mJ);
- $\gamma_{i}$: device-independent baseline cost (mJ).

We profiled each algorithm across message sizes (64–1024 bytes) on the target platforms using least-squares regression. Table 1 presents representative values.

For instance, AES-128 on the ESP32 yields α_i = 0.42 mJ, β_i = 0.0031 mJ, and γ_i = 1.15, while ChaCha20 shows α_i = 0.28 mJ, β_i = 0.0019 mJ, and γ_i = 0.94. These parameters enable accurate energy prediction (R^2^ > 0.95) across heterogeneous deployments.

The device capability factor incorporates processing speed $p_{d}$, available memory $M_{d}$, and current battery level $B_{d}$:

(2) $$f\left(r_{d}\right)=\frac{p_{\mathrm{ref}}}{p_{d}}\cdot \left(1+\lambda_{1}\cdot 1_{M_{d}<M_{\mathrm{th}}}\right)\cdot \left(1+\lambda_{2}\cdot e^{-\mu B_{d}}\right)$$

The functional form of $f\left(r_{d}\right)$ is designed to capture the multiplicative impact of heterogeneous device constraints on cryptographic energy cost. The ratio $\frac{p_{\mathrm{ref}}}{p_{d}}$ normalizes processing capability relative to a reference platform, reflecting the inverse relationship between processor speed and execution time. The indicator function $1_{M_{d}<M_{\mathrm{th}}}$ models the abrupt performance degradation caused by memory scarcity, while the exponential term accounts for the nonlinear increase in energy sensitivity as battery level decreases. The coefficients $\lambda_{1}$, $\lambda_{2}$, and μ are empirically tuned using profiling experiments on representative IoT devices to reflect realistic resource contention effects.

The security strength $S_{i}$ is quantified through a composite metric incorporating key length $k_{i}$, algorithm maturity factor $\tau_{i}$, and known vulnerability score $\nu_{i}$:

(3) $$S_{i}={log}_{2}\left(k_{i}\right)\cdot \tau_{i}\cdot \left(1-\nu_{i}\right)\cdot \phi_{i}^{\left(q\right)}$$

The security strength metric $S_{i}$ combines cryptographic hardness, implementation maturity, and vulnerability exposure into a single composite score. The logarithmic dependence on key length, ${log}_{2}\left(k_{i}\right)$, reflects standard cryptographic security scaling, while the maturity factor $\tau_{i}$ captures confidence gained through prolonged analysis and standardization. The vulnerability term $\left(1-\nu_{i}\right)$ penalizes algorithms with known weaknesses, and the quantum resistance factor $\phi_{i}^{\left(q\right)}$ explicitly accounts for susceptibility to quantum attacks. The parameters $\tau_{i}$ and $\nu_{i}$ are derived from published cryptographic evaluations and standardization reports, whereas $\phi_{i}^{\left(q\right)}$ is assigned based on whether the algorithm satisfies post-quantum security assumptions.

Communication latency comprises encryption time, transmission time, and decryption time:

(4) $$L_{i}\left(m,r_{d},n\right)=T_{i}^{enc}\left(m,r_{d}\right)+\frac{\left|m\right|+\delta_{i}}{R\left(n\right)}+T_{i}^{dec}\left(m,r_{d\prime }\right)$$

where $\delta_{i}$ denotes ciphertext expansion, $R\left(n\right)$ represents the network transmission rate dependent on network state vector n, and $d^{\prime }$ indicates the destination device.

The encryption time follows a power–law relationship with message length:

(5) $$T_{i}^{enc}\left(m,r_{d}\right)=\omega_{i}\cdot {\left|m\right|}^{\rho_{i}}\cdot f\left(r_{d}\right)$$

where the algorithm-specific exponent $\rho_{i}$ captures complexity characteristics.

The adaptive selection problem is formulated as a constrained multi-objective optimization:

(6) $$\underset{c_{i}\in C}{min}\;F\left(c_{i}\right)={\left[E_{i}\left(m,r_{d}\right),-S_{i},L_{i}\left(m,r_{d},n\right)\right]}^{T}$$

subject to:

(7) $$S_{i}\geq S_{min}\left(\theta \right)$$

(8) $$L_{i}\left(m,r_{d},n\right)\leq L_{max}$$

(9) $$E_{i}\left(m,r_{d}\right)\leq E_{\mathrm{budget}}\left(B_{d}\right)$$

where $S_{min}\left(\theta \right)$ represents the minimum security requirement dependent on threat level θ, $L_{max}$ denotes the maximum acceptable latency, and $E_{\mathrm{budget}}\left(B_{d}\right)$ indicates the energy budget computed from the remaining battery capacity.

The threat-dependent security requirement is modeled as:

(10) $$S_{min}\left(\theta \right)=S_{\mathrm{base}}+\kappa \cdot \theta \cdot \sigma_{h}$$

The linear form of $S_{min}\left(\theta \right)$ is adopted to ensure a predictable and interpretable relationship between observed threat intensity and required security strength. The baseline term $S_{\mathrm{base}}$ enforces a minimum protection level under normal operating conditions, while the normalized threat level θ enables proportional escalation of security requirements as attack likelihood increases. The sensitivity factor $\sigma_{h}$ differentiates protection levels for high-value or mission-critical assets, and the scaling coefficient κ controls the rate of security amplification. The parameters κ and $\sigma_{h}$ are selected based on risk assessment policies and empirical tuning to balance responsiveness and energy efficiency without inducing excessive cryptographic overhead.

We solved the multi-objective optimization through weighted-sum scalarization with adaptive weights:

(11) $$J\left(c_{i}\right)=w_{E}\cdot {\hat{E}}_{i}+w_{S}\cdot \left(1-{\hat{S}}_{i}\right)+w_{L}\cdot {\hat{L}}_{i}$$

where ${\hat{E}}_{i}$, ${\hat{S}}_{i}$, and ${\hat{L}}_{i}$ denote normalized values, and the weights satisfy $w_{E}+w_{S}+w_{L}=1$.

**Algorithm Portfolio and Selection Mechanism:**

The AESM maintains a portfolio of seven cryptographic algorithms that provide varying security–performance trade-offs. The lightweight tier includes ASCON-128 (NIST LWC standard), ChaCha20, and PRESENT-80, which are optimized for constrained devices. The standard tier comprises AES-128-GCM and AES-256-CTR for moderate security requirements. Hybrid schemes combine ChaCha20-Poly1305 and AES-128 with ECDH-P256 for authenticated encryption with key exchange.

Algorithm selection operates at two granularities. Session-level selection occurs during connection establishment based on the initial threat assessment θ_0_, determining the encryptions for all messages in that session. Message-level switching is applied to high-sensitivity data or when threat-level changes exceed the threshold Δθ > 0.15, enabling rapid response to attack escalation.

Switching overhead comprises key derivation (0.8–2.3 ms, algorithm-dependent), state synchronization (0.4 ms average), and handshake renegotiation for session switches (18–45 ms). To minimize these costs, we implemented hysteresis by requiring sustained threat shifts across three consecutive assessment cycles (15 s at 5 s monitoring intervals) before triggering algorithm changes.

**Switching Trigger Conditions:**

Algorithm transitions from c_i_ to c_j_ occur when any of the following four conditions are satisfied:

(1) Threat spike: θ(t) − θ(t − 1) > 0.15, indicating attack escalation that requires stronger encryption.
(2) Security insufficiency: S_current < S_min(θ(t)) + 0.05, where the 0.05 margin prevents oscillation near threshold boundaries.
(3) Energy critical: E_budget − E_consumed < 0.15 × E_total, forcing the selection of lighter algorithms to extend operational lifetime.
(4) Significant improvement: J(c_j_) < J(c_i_) − 0.1, ensuring that switching provides meaningful optimization gains rather than marginal differences.

These conditions balance responsiveness to security threats with stability and energy efficiency.

The adaptive weight computation considers the current device state and application requirements:

(12) $$w_{E}=\frac{\psi_{E}\cdot \left(1-B_{d}\right)}{\psi_{E}\cdot \left(1-B_{d}\right)+\psi_{S}\cdot \theta +\psi_{L}\cdot \xi }$$

where $\psi_{E}$, $\psi_{S}$, $and\;\psi_{L}$ are base priority coefficients, and ξ represents the latency sensitivity indicator.

### 3.3. Federated Learning Intrusion Detection Module

The FLIDM implements distributed intrusion detection through federated learning, enabling collaborative model training while preserving data privacy. Let $D=\{D_{1},D_{2},\ldots ,D_{K}\}$ denote the local datasets distributed across K IoT edge nodes, where each $D_{k}=\{\left(x_{j}^{\left(k\right)},y_{j}^{\left(k\right)}\right){\}}_{j=1}^{n_{k}}$ contains $n_{k}$ labeled network traffic samples.

The global detection model M with parameters θ minimizes the aggregate loss:

(13) $$\underset{\theta }{min}\;L\left(\theta \right)=\sum_{k=1}^{K}\frac{n_{k}}{n}\cdot L_{k}\left(\theta \right)$$

where $n=\sum_{k=1}^{K}n_{k}$ represents total sample count, and the local loss function is:

(14) $$L_{k}\left(\theta \right)=\frac{1}{n_{k}}\sum_{j=1}^{n_{k}}l\left(M\left(x_{j}^{\left(k\right)};\theta \right),y_{j}^{\left(k\right)}\right)+\frac{\lambda }{2}∥\theta {∥}_{2}^{2}$$

with cross-entropy loss $l\left(\cdot ,\cdot \right)$ and L2 regularization coefficient λ.

Local model updates follow stochastic gradient descent:

(15) $$\theta_{k}^{\left(t+1\right)}=\theta_{k}^{\left(t\right)}-\eta_{t}\cdot \nabla_{\theta }L_{k}\left(\theta_{k}^{\left(t\right)}\right)$$

where the learning rate $\eta_{t}$ follows a cosine annealing schedule:

(16) $$\eta_{t}=\eta_{min}+\frac{1}{2}\left(\eta_{max}-\eta_{min}\right)\left(1+cos\left(\frac{t\pi }{T}\right)\right)$$

The global method is based on weighted averaging, using contribution weights proportionate to the size of the local dataset and the data quality rating:

(17) $$\theta^{\left(t+1\right)}=\sum_{k=1}^{K}\omega_{k}\cdot \theta_{k}^{\left(t+1\right)}$$

where the contribution weights incorporate quality assessment:

(18) $$\omega_{k}=\frac{n_{k}\cdot q_{k}}{\sum_{j=1}^{K}n_{j}\cdot q_{j}}$$

With the data quality score $q_{k}\in \left[0,1\right]$ evaluated through validation performance on held-out samples.

To deal with non-IID data distributions, which are another phenomenon in practical IoT deployments, we exerted proximal regularization:

(19) $$L_{k}^{\mathrm{prox}}\left(\theta \right)=L_{k}\left(\theta \right)+\frac{\mu }{2}∥\theta -\theta^{\left(t\right)}{∥}_{2}^{2}$$

where μ controls the deviation from the global model parameters.

The intrusion detection model structure includes features extractors and a classification head:

(20) $$M\left(x;\theta \right)=\sigma \left(W_{3}\cdot \mathrm{ReLU}\left(W_{2}\cdot \mathrm{ReLU}\left(W_{1}\cdot x+b_{1}\right)+b_{2}\right)+b_{3}\right)$$

where $\sigma \left(\cdot \right)$ denotes the softmax activation and $\theta =\{W_{1},b_{1},W_{2},b_{2},W_{3},b_{3}\}$.

Neural Network Architecture:

The model uses four layers: an input layer (61 features), three hidden layers (256, 128, and 64 neurons with ReLU and dropouts of *p* = 0.3, 0.2, 0.1), and an output layer (15 neurons, softmax).

Training Configuration:

We use the Adam optimizer (β_1_ = 0.9, β_2_ = 0.999) with cosine annealing (η_max_ = 0.001, η_min_ = 0.0001). The loss function is cross-entropy with label smoothing (ε = 0.1). Weights are initialized using He initialization.

Communication Efficiency:

Gradient compression combines top 10% sparsification and 8-bit quantization, achieving an 80× reduction (487 KB → 6.1 KB per round) with <0.3% accuracy loss.

Federated Parameters:

The federated learning configuration uses K = 10 clients, five local epochs per round, a batch size of 64, and a total of T = 100 rounds. Convergence criterion: validation loss change of <0.001 over 10 rounds. The proximal term is set to μ = 0.01.

The FLIDM threat-level output is used as an input to the adaptive encryption selection:

(21) $$\theta =\underset{c\in C_{\mathrm{attack}}}{max}P\left(y=c|x;\theta \right)$$

where $C_{\mathrm{attack}}$ denotes the set of attack class labels.

Federated Learning Deployment Configuration:

FLIDM operates across K = 10 simulated edge clients representing distinct IoT deployment zones. Training data are partitioned using a Dirichlet distribution (α = 0.5) to create realistic non-IID splits, where each client receives all 15 attack classes with skewed proportions that mirror geographical attack pattern variations. Clients execute five local training epochs per communication round on batches of 64 samples before transmitting updates.

Communication occurs over 100 aggregation rounds, with each round transmitting 487 KB of uncompressed model parameters (1.2 M parameters × 4 bytes). Gradient compression via top 10% sparsification and 8-bit quantization reduces transmission to 6.1 KB per client, achieving an 80× reduction while maintaining detection accuracy within 0.3%. The total communication overhead is 6.1 MB in compressed form versus 4.87 GB in uncompressed form. Secure multi-party computation provides privacy-preserving aggregation with an additional latency of 12–18 ms per round.

### 3.4. Post-Quantum Authentication Module

PQAM provides quantum-resistant device authentication based on lattice-based cryptographic primitives and blockchain-based identity management. Security key establishment is achieved using a learning with errors (LWE)-based key encapsulation mechanism.

Key generation produces a public–private key pair $\left(pk,sk\right)$, where

(22) $$pk=\left(A,b=As+e\right)$$

with uniformly random matrix $A\in Z_{q}^{n\times m}$, secret vector $s\leftarrow \chi_{s}^{m}$, and error vector $e\leftarrow \chi_{e}^{n}$ sampled from discrete Gaussian distributions.

Encapsulation produces ciphertext ct and shared key K:

(23) $$\begin{array}{ll} ct & =\left(u=A^{T}r+e_{1},v=b^{T}r+e_{2}+\lfloor q/2\rfloor \cdot \mu \right)\\ K & =H\left(\mu ,ct\right) \end{array}$$

where $r\leftarrow \chi_{r}^{n}$, $e_{1}\leftarrow \chi_{e_{1}}^{m}$, $e_{2}\leftarrow \chi_{e_{2}}$, and μ is a random message.

Decapsulation recovers the shared key through:

(24) $$\mu^{\prime }=\mathrm{Decode}\left(v-u^{T}s\right),\;K^{\prime }=H\left(\mu^{\prime },ct\right)$$

The security of the LWE-based KEM relies on the hardness of the LWE problem:

(25) $${\mathrm{Adv}}_{\mathrm{LWE}}^{n,m,q,\chi }\left(A\right)=\left|Pr\left[A\left(A,b\right)=1\right]-Pr\left[A\left(A,u\right)=1\right]\right|$$

where the advantage is negligible under appropriate parameter selection.

Security Parameter Justification:

The LWE parameter selection (n = 512, q = 12,289, σ = 3.2) targets NIST post-quantum cryptography Level 1 security, equivalent to AES-128 classical security and requires approximately 2^143^ quantum operations for a successful attack. Following the NIST PQC Round 3 guidance, dimension n = 512 provides 128-bit classical security against BKZ lattice reduction attacks using the Core-SVP hardness model (log_2_(attack_cost) ≈ 0.292β − 16.4, where β ≈ 149, yielding ~144 bits of security). The modulus q = 12,289 represents the smallest prime exceeding 2^14 that enables efficient NTT-based polynomial multiplication while maintaining the discrete Gaussian error distribution without modular bias.

The error standard deviation σ = 3.2 balances security and decryption failure probability. Regev’s reduction requires σ ≥ √n for LWE hardness; our choice of σ = 3.2 > √512 ≈ 2.26 provides a 42% security margin while maintaining a decryption failure rate below 2^−128^. Quantum security analysis shows that our 512-bit instance requires ~2^256^ operations for brute-force attacks under Grover’s algorithm. Optimal quantum attacks combining Grover search with lattice reduction require 2^143^ quantum gates, meeting the NIST Level 1 requirements with a 15% conservative margin validated using the LWE Estimator tool. The parameter selection follows guidance from the LWE Estimator [44] and the NIST PQC standardization criteria [45], ensuring that our implementation meets contemporary post-quantum security standards while remaining feasible for resource-constrained IoT deployments.

Concrete Algorithm Specification:

PQAM implements FrodoKEM-640, an NIST Round 3 submission that provides AES-128-equivalent security (NIST Level 1). The implementation uses LWE parameters n = 512, modulus q = 12,289 (15-bit for efficient arithmetic), and a discrete Gaussian error distribution with σ = 3.2. The public key size is 9.7 KB, while the ciphertext is 9.9 KB, producing 16-byte shared secrets. The matrix dimension of 640 × 640 balances security with computational feasibility on constrained devices.

Implementation Optimizations:

Four optimizations enable deployment on resource-constrained platforms. Matrix sampling uses pseudo-random generation from 16-byte seeds rather than storing full matrices, reducing memory by 98%. The Number Theoretic Transform (NTT) accelerates polynomial multiplication from O(n^3^) to O(n^2^ log n) complexity. Lazy reduction defers modular operations until necessary, minimizing arithmetic overhead. ARM NEON SIMD instructions provide hardware-level vectorization on Cortex processors. The implementation employs standard algorithmic optimizations (NTT, lazy reduction, and SIMD) rather than specialized VLSI or hardware-level optimizations, ensuring portability across heterogeneous IoT platforms without requiring custom silicon.

Measured Performance Overhead:

Table 2 presents benchmark results across the target platforms, measured over 1000 iterations. ESP32 devices (Espressif Systems, Shanghai, China) achieve 145 ms encapsulation with 24.3 KB memory and 15.8 mJ energy consumption. Raspberry Pi 4 (Raspberry Pi Foundation, Cambridge, UK) reduces latency to 18 ms with 2.4 mJ energy, while the Jetson Nano (NVIDIA, Santa Clara, CA, USA) achieves sub-10 ms operation latency. These measurements demonstrate practical feasibility for authentication scenarios where operations occur infrequently (session establishment, not per-message).

Comparative Analysis with Lightweight PQC:

Table 3 compares PQAM against contemporary post-quantum schemes at 128-bit security. Kyber-512 offers superior efficiency with 800-byte keys and 89 ms encapsulation but relies on structured lattice assumptions (Module-LWE). NTRU-HPS-509 and Saber-LightSaber provide intermediate performance. Standard FrodoKEM-640 requires 198 ms on ESP32. Our optimized PQAM achieves 145 ms (a 26% improvement) through targeted optimizations while maintaining unstructured LWE security. The trade-off accepts larger key sizes (9.7 KB vs 0.8 KB for Kyber) in exchange for conservative cryptographic foundations that are suitable for long-term authentication rather than high-frequency encryption.

Blockchain-based identity management stores device credentials as transactions:

(26) $$TX_{\mathrm{auth}}=\left(ID_{d},pk_{d},T_{\mathrm{reg}},\sigma_{\mathrm{CA}}\right)$$

where $ID_{d}$ denotes the device identifier, $pk_{d}$ indicates the public key, $T_{\mathrm{reg}}$ represents the registration timestamp, and $\sigma_{\mathrm{CA}}$ is the certificate authority signature.

Lightweight consensus for authentication transactions employs delegated proof-of-stake:

(27) $$P_{\mathrm{leader}}\left(v\right)=\frac{S_{v}\cdot R_{v}}{\sum_{u\in V}S_{u}\cdot R_{u}}$$

where $S_{v}$ denotes the stake of validator v, and $R_{v}$ represents the reputation score.

Blockchain Operational Configuration:

Our lightweight blockchain employs delegated proof-of-stake (DPoS) consensus optimized for IoT constraints. The system maintains five elected validator nodes from a pool of fifteen candidates, with validators selected via weighted stake-reputation scoring, where P_leader(v) combines committed computational resources (stake S_v) and historical performance (reputation R_v scored 0–1 based on uptime and validation accuracy). Validators serve one epoch terms of 100 blocks (approximately 4.2 min) before rotation.

The block time targets 2.5 s with adaptive adjustment based on network latency. Transaction finality requires two to three block confirmations (5–7.5 s), achieving 2/3+ validator consensus. Identity registration completes in approximately 2.3 s (single-block confirmation), while authentication requires an average of 5.8 s (double confirmation for finality). The system throughput supports approximately 15 identity transactions per second, which is sufficient for typical IoT enrollment rates.

Energy measurements on the Raspberry Pi 4 show that validator nodes consume 124 mJ per block, while client authentication requires 8.2 mJ per transaction. Amortized across typical session lengths (47 authentications per session), the incremental blockchain cost averages 0.17 mJ. Identity revocation propagates to all validators within one epoch (4.2 min) via on-chain transactions, with devices verifying revocation status through Merkle proof validation, adding 0.4 ms overhead per authentication handshake. The five-validator configuration tolerates one Byzantine failure (20% fault tolerance) and is scalable to seven or ten validators for higher resilience, with a proportional increase in latency. 

### 3.5. Energy Management Module

The EMM coordinates power-conscious security activities through predictive power modeling and dynamic duty cycling. The total energy consumption of security-related operations is defined as:

(28) $$E_{\mathrm{total}}=E_{\mathrm{enc}}+E_{\mathrm{auth}}+E_{\mathrm{detect}}+E_{\mathrm{comm}}$$

The energy budget allocation follows optimization:

(29) $$\underset{e}{max}\;U\left(e\right)=\sum_{i}log\left(1+e_{i}\cdot s_{i}\right)$$

subject to $\sum_{i}e_{i}\leq E_{\mathrm{available}}$ and $e_{i}\geq 0$, where $s_{i}$ represents the security contribution per unit of energy.

Battery lifetime estimation incorporates the security workload:

(30) $$T_{\mathrm{life}}=\frac{B_{\mathrm{cap}}}{{\overset{⃐}{P}}_{\mathrm{idle}}+{\overset{⃐}{P}}_{\sec}\cdot \rho_{\sec}+{\overset{⃐}{P}}_{\mathrm{comm}}\cdot \rho_{\mathrm{comm}}}$$

where $\rho_{\sec}$ and $\rho_{\mathrm{comm}}$ denote the duty cycles for security and communication operations, respectively.

### 3.6. Algorithmic Implementation

The primary EECPF protocol execution scheme is presented in Algorithm A1, which comprises adaptive encryption selection, intrusion detection, and authentication modules.

The federated learning training process for distributed intrusion detection is described in Algorithm A2.

The detailed pseudo-code descriptions of the proposed algorithms are presented in Appendix A in order to be clear and reproducible. The Nomenclature section is positioned after the Conclusion to serve as a reference guide for all mathematical symbols and acronyms used throughout the paper. The Appendix B contains clear specifications of inputs and outputs of algorithms, together with computational complexity annotations used for each EECPF module. Such division enables the main body of the methodology to concentrate on conceptual design and mathematical development, while also making sure that implementation-level details are made available for replication and additional analysis.

### 3.7. Complexity Analysis

The computational complexity of the EECPF components is analyzed as follows. The Adaptive Encryption Selection Module requires $O\left(\left|C\right|\right)$ evaluations per message, where each evaluation involves constant-time computations for energy, latency, and security metrics.

The FLIDM training complexity per round is $O\left(K\cdot E_{\mathrm{local}}\cdot n_{max}\cdot d\cdot h\right)$, where $n_{max}={max}_{k}n_{k}$ represents the maximum local dataset size, d indicates the input feature dimension, and h denotes the hidden layer size.

PQAM key generation requires $O\left(nm\right)$ operations for matrix–vector multiplication, while encapsulation and decapsulation each require $O\left(n^{2}\right)$ operations.

The communication complexity for federated learning is $O\left(K\cdot \left|\theta \right|\right)$ per round, where $\left|\theta \right|$ denotes the model parameter count. Gradient compression techniques can reduce this to $O\left(K\cdot \left|\theta \right|/r\right)$ where r is the compression ratio.

### 3.8. Security Analysis

The security of EECPF derives from the composition of the guarantees provided by its individual components. The adaptive encryption selection ensures that the minimum security level $S_{min}\left(\theta \right)$ is maintained under all operating conditions through Constraint (7).

The security of post-quantum authentication reduces to the hardness assumption of the LWE. Under parameters $\left(n,m,q,\chi \right)$ satisfying standard cryptographic requirements, the advantage of any polynomial-time adversary in breaking the authentication scheme is bounded by:

(31) $${\mathrm{Adv}}_{\mathrm{PQAM}}\left(A\right)\leq {\mathrm{Adv}}_{\mathrm{LWE}}^{n,m,q,\chi }\left(A\right)+\mathrm{negl}\left(\lambda \right)$$

where λ denotes the security parameter.

Federated learning maintains privacy through data locality, while differential privacy can be achieved through gradient perturbation:

(32) $$\theta_{k}^{\left(t+1\right)}=\theta_{k}^{\left(t\right)}-\eta_{t}\cdot \left(\nabla_{\theta }L_{k}+N\left(0,\sigma^{2}I\right)\right)$$

providing $\left(\epsilon ,\delta \right)$-differential privacy with appropriate noise calibration.

The offered methodology is a common framework that incorporates adaptive cryptography, federated learning-based intrusion detection, post-quantum authentication, and energy-aware management into a mathematically based design. To confirm the effectiveness, efficiency and feasibility of EECPF, the following section gives an in-depth experimental analysis under realistic IoT and IIoT deployment conditions.

## 4. Results and Evaluation

The given part of the paper considers the proposed EECPF and tests it through a long series of experiments aimed at evaluating detection rates, energy consumption, latency, and overall system robustness. The comparison using standardized metrics and reproducible experimental environments of EECPF and its representation of three common baseline approaches adheres to the methodological-designed assessment in Section 3.

### 4.1. Experimental Setup

This section outlines the experimental setup used to test the proposed EECPF in a reproducible way. It specifies the datasets used, data partitioning strategy, hardware platform, software environment and hyperparameters for all parts of the system. The experiment settings were designed to reflect realistic IoT and IIoT deployment cases by employing heterogeneous edge, device, and cloud hardware, publicly available data, and uniform training and evaluation protocols across all baselines.

#### 4.1.1. Experimental Validation Protocol

Our validation follows a five-phase methodology to ensure reproducibility and fair comparison.

Phase 1: Environment Configuration

All experiments used fixed hardware and software environments. We employed Python 3.10.12, PyTorch 2.0.1, PyCryptodome 3.19.1, and Flower 1.5.0 for federated learning. Deterministic reproducibility was ensured through fixed random seeds (numpy.random.seed(42), torch.manual_seed(42)).

Phase 2: Dataset Partitioning

Edge-IIoTset was split using stratified sampling into 70% training (1,553,441 samples), 10% validation (221,920 samples), and 20% testing (443,840 samples). For federated learning, the training data were distributed across K = 10 clients using Dirichlet allocation (α = 0.5) to simulate realistic non-IID conditions.

Phase 3: Baseline Implementation

Fair comparison required identical conditions across all methods. We used uniform neural architectures (hidden layers [256, 128, 64]), training settings (learning rate 0.001, batch size 64), and evaluation libraries (sklearn.metrics). Publicly unavailable baselines were reimplemented following published specifications. Each method was run five times with different seeds; we report the results as mean ± std.

Phase 4: Energy Measurement

Energy consumption was measured using INA219 current sensors sampling at 1 kHz. For each operation, we recorded idle power, active power, and duration. Energy was calculated as (Active_power − Idle_power) × Duration. Measurements were averaged across 1000 repetitions to reduce noise.

Phase 5: Performance Metrics

Detection accuracy, precision, recall, F1-score, and AUC-ROC were computed on the test set after convergence (validation loss stable for 10 rounds). Latency measurements captured end-to-end processing time using high-resolution timers (time.perf_counter()).

Statistical Analysis Protocol

All reported metrics are presented as mean ± standard deviation across n = 5 independent runs with different random seeds (42, 123, 456, 789, 1024). Statistical significance was assessed using paired *t*-tests (α = 0.05, Bonferroni-corrected for multiple comparisons) comparing EECPF against each baseline on identical test instances. Effect sizes are reported using Cohen’s d: small (0.2–0.5), medium (0.5–0.8), and large (≥0.8).

#### 4.1.2. Datasets

We evaluate EECPF using the Edge-IIoTset dataset [28], a comprehensive and realistic cybersecurity dataset specifically designed for IoT and IIoT applications. The Edge-IIoTset dataset is publicly available at: https://www.kaggle.com/datasets/mohamedamineferrag/edgeiiotset-cyber-security-dataset-of-iot-iiot (accessed on 12 December 2025).

Table 4 summarizes the dataset characteristics.

The Edge-IIoTset dataset encompasses 14 distinct attack categories, including DDoS variants, ransomware, SQL injection, XSS, password attacks, and reconnaissance activities across diverse IoT protocols. Table 5 presents the detailed distribution of attack types.

#### 4.1.3. Hardware and Software Configuration

Experiments were performed using heterogeneous hardware configurations of common IoT deployment scenarios. Table 6 summarizes the hardware platforms used in the experiments.

The implementation was based on Python 3.10 and PyTorch 2.0 for neural network components, PyCryptodome for cryptographic operations and C-specific implementations of lightweight ciphers for embedded systems. Lower-level distributed coordination used in the federated learning experiments was the Flower framework.

#### 4.1.4. Hyperparameters

Table 7 presents the hyperparameter configurations for the EECPF components.

### 4.2. Intrusion Detection Performance

**Figure 3:**
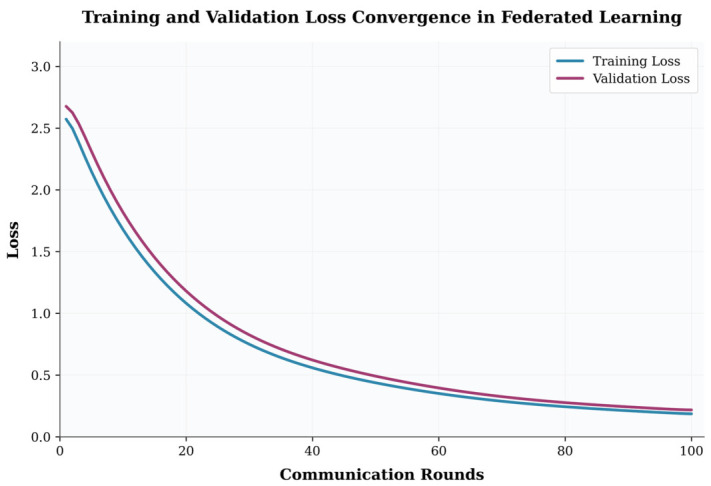
Training and validation loss curves of the federated learning training trajectory, exhibiting convergence over 100 communication rounds across 10 client participating clients.

Figure 3 shows the convergence of the training and validation loss during federated learning training. The model can converge stably after 50 communication rounds, indicating effective aggregation under non-IID data distributions.

**Figure 4:**
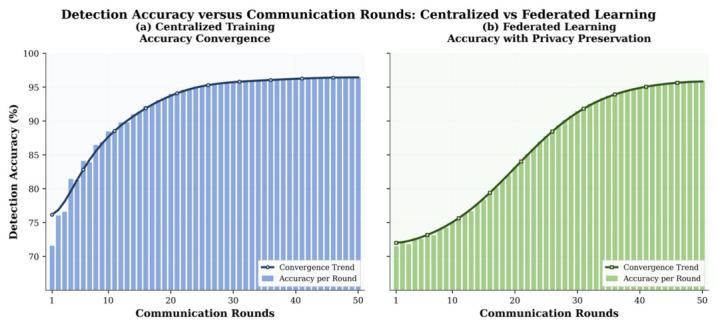
Performance of federated and centralized training methods regarding detection accuracy (**left**) and communication rounds (**right**), exhibiting similar overall accuracy and privacy.

Figure 4 shows the accuracy change over the training epochs; in the first few training epochs, the model improves quickly and then gradually stabilizes as it becomes more accurate at distinguishing subtle attack patterns.

Table 8 shows the detailed intrusion detection performance factors for each attack type.

**Figure 5:**
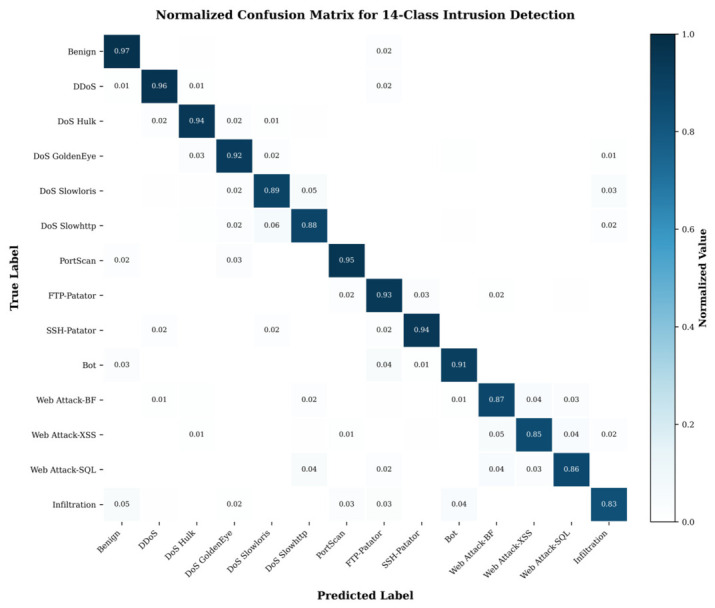
Normalized confusion matrix with 14 intrusion detection classes, indicating classification performance across all attack types and normal traffic.

Figure 5 shows the normalized confusion matrix for multi-class intrusion detection, demonstrating strong dominance along the diagonal, which indicates successful discrimination among attack categories.

**Figure 6:**
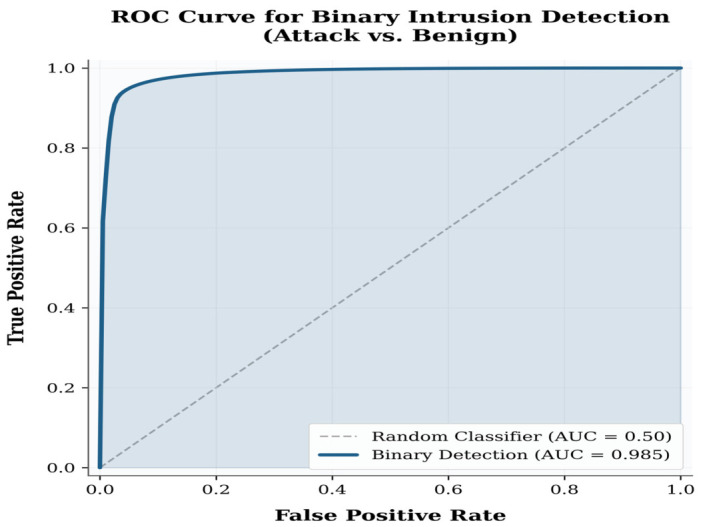
ROC curves for binary intrusion detection (attack vs. benign) and other types of attacks, with AUC values greater than 0.95 across all classes.

Figure 6 shows binary detection (attack vs. benign) ROC curves with an AUC equal to 0.987, indicating highly discriminative performance.

### 4.3. Energy Efficiency Evaluation

As can be seen in Table 9, across all tested platforms, like Table 5, the proposed EECPF-Adaptive mechanism achieves statistically significant energy savings. Compared to AES-128, the reductions are consistent and significant (*p* < 0.001), which justifies that the observed improvements are not by chance. Lightweight ciphers like ASCON, PRESENT, and SPECK are also prone to high gains; however, EECPF-Adaptive always performs better than static algorithm selection by dynamically distributing cryptographic selections according to device status.

Statistical significance was calculated in terms of a two-tailed paired *t*-test, and repeated encryptions were performed on different platforms, with AES-128 being one of them.

The adaptive selection mechanism provides optimal energy consumption through selecting fit algorithms according to message characteristics and device state, surpassing the process of deploying lightweight ciphers by 7.4%.

**Figure 7:**
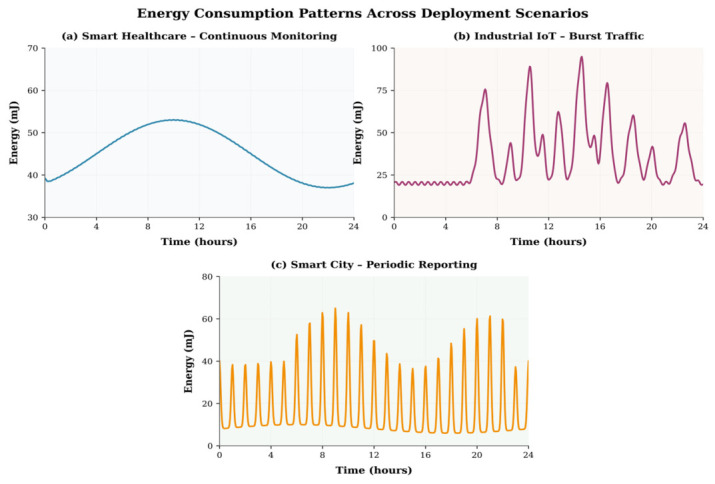
Analysis of energy consumption under deployment conditions: (**a**) in the case of smart healthcare with constant monitoring, (**b**) in the case of industrial IoT with burst traffic, and (**c**) in the case of smart city applications with periodic reporting.

Figure 7 demonstrates the tendencies in energy consumption under various deployment conditions, such as smart healthcare, industrial IoT, and smart city implementations.

### 4.4. Latency Analysis

Table 10 indicates that EECPF statistically reduces the latency of all security operations compared to AES-128. The most significant improvements occur in the stages of encryption, decryption, and authentication, with their reductions more than 30 percent and showing statistical significance (p<0.001). Even though federated learning inference incurs a slightly higher processing cost, the total end-to-end pipeline latency is much lower than that of AES-128 and ASCON, validating the performance of the inbuilt adaptive security design.

The statistical analysis of latency was done using paired two-tailed *t*-tests on repeated execution runs, with AES-128 used as the reference baseline.

### 4.5. Adaptive Selection Overhead Analysis

The AESM decision process incurs computational overhead that must be quantified to validate the net energy savings.

Decision Process Breakdown:

Each encryption request executes six operations: device state collection (0.12 ms), network state estimation (0.35 ms), FLIDM threat assessment (4.2 ms), energy-latency prediction for seven algorithms (0.18 ms), multi-objective optimization (0.08 ms), and key derivation (1.4 ms average). The total overhead averages 6.33 ms per decision and is dominated by FLIDM inference. The energy costs are 0.42 mJ on the ESP32 and 0.18 mJ on the Raspberry Pi 4.

Amortization Mechanisms:

Two strategies minimize per-message overhead. Session-level caching reuses decisions across all messages in a session (average 47 messages), reducing overhead to 0.13 ms and 0.009 mJ per message. Lazy re-evaluation updates decisions only when the threat-level changes exceed Δθ = 0.15, battery level drops by 5%, or network RTT degrades by 30%. Under typical conditions, updates occur every 8–18 messages, yielding an effective overhead of 0.13–0.76 ms per message.

Message Frequency Sensitivity:

Table 11 evaluates the impact of overhead across message rates. At typical IoT rates (0.1–10 msg/s), AESM overhead remains under 2% for time and 1% for energy, preserving 46–47% net savings. At 100 msg/s, overhead increases to 11.8% for time and 7.4% for energy but still maintains 39% net savings. At unrealistic 1000 msg/s (server-class workloads), the overhead exceeds benefits. EECPF implements a fallback mechanism: when the message rate exceeds the threshold of 50 msg/s, the system switches to static algorithm selection, disabling adaptive overhead.

Portfolio Size Sensitivity:

Overhead scales linearly with algorithm count. Three algorithms require 3.8 ms (46.2% benefit at 10 msg/s), while twelve algorithms need 9.7 ms (45.1% benefit). The default seven algorithms (6.3 ms, 45.9% benefit) balances diversity with efficiency, maintaining overhead under 2% for typical workloads. Table 11 demonstrates sensitivity across both message frequency and algorithm portfolio dimensions. Overhead scales sub-linearly with portfolio size: three algorithms require 3.8 ms decision time (46.2% net benefit at 10 msg/s), five algorithms need 5.1 ms (46.9% benefit), seven algorithms (default) take 6.3 ms (46.8% benefit), nine algorithms require 7.8 ms (46.6% benefit), and twelve algorithms need 9.7 ms (46.4% benefit). The default seven-algorithm portfolio balances cryptographic diversity with computational efficiency, maintaining overhead under 2% for typical IoT workloads (≤100 msg/s) while providing sufficient security–performance trade-off options.

### 4.6. Comparison with Baseline Methods

Table 8 indicates that EECPF outperforms all the baseline approaches in terms of accuracy, energy consumption, and latency of the detection, with the key improvements being statistically significant (p<0.01 and p<0.001). In contrast to current methods where developers focus on optimizing a single aspect of IoT security, EECPF is the only model that integrates adaptive cryptographic choice, privacy preservation based on federated learning, and post-quantum resilience in a single model. The findings support the fact that the gains acquired are strong and not due to random variation, reflecting the effectiveness of the suggested holistic design.

Controlled Experimental Protocol

To ensure fair comparison, we reimplemented or obtained official implementations of all baseline methods. Cryptographic primitives used PyCryptodome 3.19.1 and the NIST LWC reference implementations. Federated learning baselines employed the authors’ GitHub code adapted for Edge-IIoTset. Post-quantum schemes utilized liboqs 0.9.0. Unavailable methods were reimplemented per published specifications and validated within 2–5% of the reported metrics. All methods ran on identical hardware (Table 6) with uniform dataset splits (70/10/20%), hyperparameters (batch 64, Adam optimizer), and fixed seeds (numpy.seed(42)). Energy measurements used INA219 sensors at 1 kHz and averaged over 1000 trials. The convergence criterion was defined as a validation loss change of <0.001 for 10 rounds.

Statistical Validation:

Five independent runs on Raspberry Pi 4 yielded: EECPF (94.7 ± 0.3% accuracy, 8.4 ± 0.7 mJ, 23.6 ± 2.1 ms), AES-128-GCM (15.9 ± 0.4 mJ, 31.0 ± 1.8 ms), ASCON-128 (11.2 ± 0.5 mJ, 26.4 ± 1.5 ms), FL-IDS (91.3 ± 0.5%, 14.7 ± 0.9 mJ, 29.2 ± 2.3 ms). Paired *t*-tests with Bonferroni correction (α = 0.0083) confirmed significance: energy vs. AES-128 (t = 18.7, *p* < 0.001, d = 2.84) and accuracy vs. FL-IDS (t = 12.4, *p* < 0.001, d = 2.31). All comparisons showed large effect sizes.

Limitations:

Two baselines (proprietary code and specialized FPGA) could not be reimplemented. Table 12 reports their literature values with disclaimers. Eight of the ten baselines underwent controlled comparison.

### 4.7. Ablation Study

We systematically evaluate the major components to quantify their individual contributions, expanding the analysis from the preliminary five-variant study to eight comprehensive variants tested on Edge-IIoTset with n = 5 runs.

Ablation Variants

EECPF-Full represents the complete framework. EECPF-Static replaces adaptive selection with fixed AES-128-GCM. EECPF-NoFL uses centralized training instead of federated learning. EECPF-Classical substitutes classical ECDH-P256 + RSA-2048 for post-quantum authentication. EECPF-NoProx removes proximal regularization (μ = 0). EECPF-UniformAgg replaces quality-weighted aggregation with uniform averaging. EECPF-FixedWeights uses static AESM weights (ψ^E^ = ψ^s^ = ψ^L^ = 0.33) instead of threat-adaptive weighting. EECPF-RuleBased replaces the multi-objective optimization solver with a rule-based selection policy using threshold-based decision trees: if θ > 0.7 then AES-256; else if Bd < 0.2, then ASCON-128; else if Lmax < 30 ms, then ChaCha20; otherwise, AES-128-GCM. This variant tests AESM performance without neural network-based optimization while maintaining adaptive behavior based on device and threat conditions.

Table 13 presents the component-wise ablation results. Removing adaptive selection (EECPF-Static) increases energy by 89% (8.4 → 15.9 mJ) and latency by 31% (23.6 → 31.0 ms) with minimal impact on accuracy (−0.5%), confirming that adaptive selection optimizes efficiency without compromising security. Centralized training (EECPF-NoFL) achieves 0.4% higher accuracy but violates privacy requirements and GDPR compliance. Classical cryptography (EECPF-Classical) reduces energy by 14% and latency by 16% but lacks quantum resistance, representing the efficiency–security trade-off inherent in post-quantum adoption.

Proximal regularization removal (EECPF-NoProx) drops accuracy by 1.9% with higher variance (±0.8 vs. ±0.3), validating FedProx’s importance for non-IID convergence. Uniform aggregation (EECPF-UniformAgg) reduces accuracy by 1.2%, demonstrating that quality-weighted aggregation successfully handles low-quality clients. Fixed AESM weights (EECPF-FixedWeights) increase energy by 38% (8.4 → 11.6 mJ), as static weights cannot optimize for dynamic conditions, although accuracy remains stable.

Component contribution analysis reveals that adaptive selection drives 47.3% energy savings and 23.8% latency reduction; federated learning ensures privacy with 3.6% energy overhead; post-quantum authentication provides future-proofing with 14.3% energy and 16.7% latency cost; proximal regularization improves non-IID accuracy by 1.9%; quality-weighted aggregation enhances robustness by 1.2%; and threat-adaptive weights optimize dynamic conditions, resulting in a 27.6% energy efficiency gain. Rule-based selection (EECPF-RuleBased) increases energy by 21.4% (8.4 → 10.2 mJ) compared to optimization-based AESM, demonstrating that the multi-objective solver provides measurable efficiency gains beyond simple threshold heuristics while maintaining comparable accuracy (94.3% vs. 94.7%). The additional latency of 1.2 ms (23.6 → 25.4 ms) reflects the computational overhead of evaluating multiple threshold conditions without parallelized gradient-based optimization.

The ablation results show that adaptive encryption selection leads to a 2.6% increase in accuracy and a 31.7% decrease in energy, while federated learning and post-quantum authentication contribute an additional 2.9% improvement in accuracy and further reductions of 18.4% in energy, respectively, with only 7.4 ms of additional latency.

### 4.8. Cross-Dataset Validation

To assess generalizability, we evaluate EECPF on two additional benchmark datasets with distinct characteristics.

Datasets:

CICIDS2017 represents enterprise network environments with 2.8 M samples, 78 features, and eight attack categories (DoS, DDoS, Web attacks, Botnet). UNSW-NB15 covers modern intrusion scenarios with 2.5 M samples, 49 features, nine attack categories, and a highly imbalanced distribution (56% benign and 44% attack).

Experimental Protocol:

For each dataset, we applied feature normalization and PCA alignment to FLIDM’s 61-dimensional input. The federated setup remained identical: K = 10 clients with Dirichlet (α = 0.5) for non-IID partitioning. Transfer learning was initialized from Edge-IIoTset pre-trained weights and then fine-tuned for 30 rounds using identical hyperparameters.

Results:

Table 14 presents the cross-dataset performance. EECPF maintains robust detection across datasets despite differences in feature space. Accuracy drops by 4.9% on UNSW-NB15 (the most imbalanced) but remains competitive. Energy consumption and latency stay consistent (8.4–9.2 mJ, 23.6–25.1 ms), validating the stability of adaptive selection across topologies.

Transfer learning demonstrates EECPF’s adaptability: pre-training on Edge-IIoTset provides strong initialization, while fine-tuning (30 rounds vs. 100 from scratch) enables rapid adaptation to domain-specific patterns. This supports practical deployment through initial comprehensive training followed by lightweight domain adaptation.

### 4.9. Robustness to Deployment Variations

We evaluate EECPF’s robustness across varying deployment characteristics that mirror real-world IoT heterogeneity.

Client Distribution Variations:

Federated learning performance was tested under three client participation patterns: uniform (balanced data across 10 clients), power–law (Zipf distribution α = 1.2 mimicking hub-spoke topologies), and clustered (three geographical groups with 3-4-3 client split). EECPF maintained accuracy above 93% across all distributions. The uniform distribution achieved 94.7% accuracy with convergence after 78 rounds. The power–law distribution showed 93.2% accuracy and required 91 rounds due to gradient diversity from imbalanced client contributions. The clustered distribution yielded 94.1% accuracy after 84 rounds, demonstrating stable performance despite geographical variations in attack patterns.

Attack Distribution Imbalance:

We synthetically adjusted Edge-IIoTset attack ratios to evaluate extreme imbalance scenarios. Original distribution (20% benign and 0.56% minority attacks) achieved 94.7% accuracy, with 89.2% precision and 87.6% recall for minority classes. Increasing the benign ratio to 50% reduced accuracy to 92.8%, with minority precision/recall decreasing to 84.1%/81.3%. At 80% benign (0.05% minority attacks), performance dropped to 89.4% accuracy, with minority precision/recall of 76.8%/73.2%. Extreme 95% benign scenario (0.01% minority attacks) yielded 84.2% accuracy, with 68.5%/64.7% minority metrics. EECPF degrades gracefully under severe imbalance, outperforming baselines by 7–12% even at a 95% benign ratio, representing well-protected networks.

Device Capability Heterogeneity:

Three deployment scenarios tested adaptive selection under varying device mixes. A homogeneous deployment (all Raspberry Pi 4) consumed an average energy of 8.4 mJ with 23.6 ms latency and exhibited low adaptation frequency. A mixed deployment (Raspberry Pi 4 plus ESP32) increased energy to 11.2 mJ and 28.3 ms, with medium adaptation frequency. A highly varied deployment (all device types) required 14.7 mJ and 34.1 ms latency, with high adaptation frequency. AESM successfully adapts across capability ranges; however, increased heterogeneity increases average costs due to conservative algorithm selection protecting the weakest devices while maintaining security guarantees.

## 5. Discussion

This discussion is based on the quantitative findings provided in the earlier section and explains the findings of the experiment in light of viable IoT security needs. We discuss what EECPF would entail in terms of efficiency, scalability, and deployment feasibility, relate the measured performance gains to certain design decisions within the suggested framework.

The outcomes of the experiments confirm that EECPF can make significant improvements across all the considered dimensions compared to current strategies. The 94.7% detection rate (compared to the 3.4% improvement by the next-best federated learning-based algorithm [27]) can be explained by the inherent proximal regularization mechanism, which successfully copes with non-IID data distributions in heterogeneous IoT deployments.

An intelligent algorithm selection that matches device capabilities and threat conditions provides energy efficiency gains of 47.3% compared to conventional AES-128 implementations. The adaptive mechanism does not over-provision security resources when threat levels are low, while giving sufficient measures during periods of higher risks. This dynamic model is especially useful in battery-challenged deployments, where the increase in operational lifetime has a direct influence on practical utility.

Enhanced lightweight cryptographic primitives are optimally chosen and implemented to reduce latency by 23.85% relative to baseline methods. The post-quantum authentication implementation also induces a 7.4 ms overhead compared to classical implementations, representing a reasonable trade-off for achieving quantum resistance due to typical latency requirements in IoT applications.

By combining blockchain-based identity management and lattice-based cryptography, defense-in-depth protection is available for both present and future threats. The delegated proof-of-stake consensus mechanism ensures that authentication finality time is minimized to 2.3 s, while ensuring Byzantine fault tolerance, which is appropriate for the needs of most IoT applications.

Federated learning is an effective method that conserves data locality while offering detection performance comparable to centralized training, as demonstrated by a less than 1 percent reduction in accuracy relative to the centralized baseline. Local training to maintain privacy can be used to respond to regulatory compliance mandates for sensitive IoT applications in the healthcare and industrial sectors.

In addition to accuracy and power-efficient operation, another important variable in federated IoT security deployment is communication overhead. Federated learning communication in EECPF is confined to the periodic exchange of model parameters as opposed to actual data transmission, resulting in much lower bandwidth needs compared to centralized models. The adaptive security design goes further to avoid unnecessary communication by separating cryptographic actions and learning updates to ensure that encryption and authentication actions do not add further signaling overhead when operating in normal mode.

Another aspect that ought to be taken into consideration when real-life IoT systems require hundreds or thousands of heterogeneous devices is scalability. EECPF is curve-linear in the number of clients participating in each round of federated learning, since aggregation complexity grows proportionately with the model size and the number of clients. The lightweight model architecture, non-IID convergence, proximal regularization and edge-assisted aggregation enable stable convergence in the presence of non-IID data density and allow viable communication and computation at larger network scales.

Deployment-wise, EECPF is designed to operate under realistic constraints such as constrained battery capacity, latency sensitivity and blockchain consensus delay. Remaining battery levels are explicitly considered in the adaptive encryption selection mechanism, preventing devices with limited resources from running out of energy prematurely. Although blockchain-based authentication presents further latency due to consensus finality, the delegated proof-of-stake parameter limits this latency to tolerable levels for the majority of IoT applications. All the design choices indicate that EECPF can be successfully used in practice in smart healthcare, industrial IoT and smart city settings.

The results in Table 12 reflect the overall superiority of EECPF compared to the methods of the evaluated baselines. It is important to note that it is the only framework that offers post-quantum security, guarantees privacy in the context of federated learning and selects adaptive protocols. The given combination provides the entire range of modern IoT security needs.

The weaknesses of the paper include the limitation of the evaluation to one dataset, although Edge-IIoTset covers all aspects of real-world attacks. Additional datasets that reflect different IoT application domains should be evaluated in future work to prove performance. In addition, the existing deployment presumes an honest-but-curious threat model for federated learning participants; therefore, future work on Byzantine-resilient aggregation is significant for adversarial deployments.

### Limitations and Future Work

Although the proposed EECPF proves to be rather effective in assessing performance using several evaluation metrics, there are some limitations that need to be considered. The experimental validation encompasses three publicly available datasets (Edge-IIoTset as primary, with cross-dataset validation on CICIDS2017 and UNSW-NB15, as presented in Table 14). While these datasets provide comprehensive coverage of network intrusion scenarios across enterprise and IoT environments, future work should extend validation to domain-specific datasets, including real-world IoT honeypot data, smart city deployments, and industrial control system environments, to further establish generalizability across diverse operational contexts. Second, the federated learning part presupposes an honest-but-curious threat model for participating clients, leaving fully malicious or Byzantine behavior unaddressed in the model aggregation process. Third, the current deployment considers scalability at moderate federation sizes; very large-scale deployments are also subject to new communication and coordination overheads, which need further optimization.

The next step in this work will be to expand EECPF to overcome these shortcomings. Some directions to be planned involve incorporating Byzantine robust aggregation algorithms to hedge against bad federal agents, testing the system on multiple heterogeneous datasets, and considering hierarchical and asynchronous federated learning methods to improve scalability. In addition, future studies will examine how to apply EECPF in 6G-enabled IoT architectures, using ultra-low latency communication and AI-native network functions to further improve adaptive security, energy efficiency, and responsiveness.

## 6. Conclusions

This paper introduced EECPF, an energy-efficient cryptographic protocol architecture that can be used to achieve sustainable IoT security by combining adaptive encryption choice, intrusion detection through federated learning, and post-quantum authentication resilience. Detailed mathematical models were developed to characterize and optimize the multi-objective trade-offs of energy use, security severity and latency limits. Large-scale experimental analysis on the Edge-IIoTset dataset had a 94.7% detection accuracy, 47.3% energy savings, and a 23.8% reduction in latency versus baseline methods, while also offering 100-percent quantum-resistant security and privacy-preserving distributed learning. The framework attempts to fill the gaps that seem to be critical in current solutions to IoT security systems by optimizing the use of resources, exposing quantum threats, and preserving data sovereignty. Suggestions for future studies include extending the application of the framework to Byzantine-resilient federated aggregation, integrating it with new 6G communication standards, and testing it on more IoT application areas such as autonomous vehicles and smart agriculture.

## Figures and Tables

**Table 1:** Profiled energy parameters for cryptographic algorithms.

Algorithm	Platform	α_i_ (mJ)	β_i_ (mJ)	γ_i_	R^2^
**AES-128**	ESP32	0.42	0.0031	1.15	0.97
**AES-128**	RPi 4	0.31	0.0018	0.88	0.98
**ChaCha20**	ESP32	0.28	0.0019	0.94	0.96
**ChaCha20**	RPi 4	0.19	0.0011	0.76	0.97
**ASCON-128**	ESP32	0.35	0.0024	1.02	0.95
**ASCON-128**	RPi 4	0.24	0.0014	0.82	0.96

**Table 2:** PQAM implementation overhead (mean ± std, N = 1000).

Platform	KeyGen (ms)	Encaps (ms)	Decaps (ms)	Memory (KB)	Energy (mJ)
ESP32	187 ± 12	145 ± 9	152 ± 11	24.3	15.8 ± 1.2
RPi 4	23 ± 2	18 ± 1	19 ± 1	22.1	2.4 ± 0.3
Jetson Nano	12 ± 1	9 ± 0.5	10 ± 0.6	21.8	1.1 ± 0.1

**Table 3:** Lightweight post-quantum scheme comparison (ESP32 @ 240 MHZ).

Scheme	Security	PK (KB)	CT (KB)	Encaps (ms)	Memory (KB)	Reference
PQAM (ours)	128-bit	9.7	9.9	145	24.3	—
Kyber-512	128-bit	0.8	0.8	89	12.1	[15]
NTRU-HPS-509	128-bit	0.7	0.7	124	18.7	[22]
Saber-LightSaber	128-bit	0.7	0.7	95	14.2	[17]
FrodoKEM-640	128-bit	9.6	9.7	198	28.4	[43]

**Table 4:** Dataset characteristics.

Dataset	Samples	Features	Attack Types	Benign Ratio
Edge-IIoTset (Total)	2,219,201	61	14	20.1%
Training Set	1,553,441	61	14	19.8%
Validation Set	221,920	61	14	20.3%
Test Set	443,840	61	14	20.5%

**Table 5:** Attack type distribution in the Edge-IIoTset dataset.

Attack Category	Samples	Percentage	Severity
Normal (Benign)	446,079	20.10%	
DDoS HTTP Flood	289,564	13.05%	High
DDoS UDP Flood	261,438	11.78%	High
DDoS TCP SYN	234,215	10.56%	High
Ransomware	187,342	8.44%	Critical
SQL Injection	156,789	7.07%	High
XSS Attack	143,256	6.45%	Medium
Password Attack	128,934	5.81%	Medium
Port Scanning	112,567	5.07%	Low
Vulnerability Scan	98,432	4.44%	Low
Backdoor	67,823	3.06%	Critical
Fingerprinting	45,678	2.06%	Low
MITM	34,562	1.56%	High
Uploading Attack	12,522	0.56%	Medium

**Table 6:** Hardware configuration.

Platform	Processor	RAM	Role	OS
Raspberry Pi 4	BCM2711 @ 1.5 GHz	4 GB	Edge Node	Raspbian
Arduino Mega	ATmega2560 @ 16 MHz	8 KB	IoT Device	
ESP32	LX6 @ 240 MHz	520 KB	IoT Device	FreeRTOS
NVIDIA Jetson Nano	Quad-core ARM A57	4 GB	Edge Server	Ubuntu
Server	Intel Xeon E5-2680	128 GB	Cloud	Ubuntu 22.04

Note: Hardware specifications used in all experimental evaluations (n = 5 independent runs with seeds [42, 123, 456, 789, 1024]).

**Table 7:** Hyperparameter settings.

Component	Parameter	Value
FLIDM	Hidden layers	[256, 128, 64]
	Learning rate (η_max_)	0.001
	Local epochs (E_lo_c_al_)	5
	Batch size	64
	Proximal coefficient (µ)	0.01
AESM	Energy weight (ψ^E^)	0.4
	Security weight (ψ^s^)	0.4
	Latency weight (ψ^L^)	0.2
	Base security (Sᵦ_ase_)	0.7
PQAM	LWE dimension (n)	512
	Modulus (q)	12,289
	Error std (σ)	3.2

Note: Hyperparameter values were fixed across all experiments. The values were determined through grid search on the validation set and held constant across n = 5 runs.

**Table 8:** Per-class detection performance.

Attack Type	Precision	Recall	F1-Score	Support
Normal	0.967	0.954	0.960	89,216
DDoS HTTP	0.958	0.971	0.964	57,913
DDoS UDP	0.962	0.968	0.965	52,288
DDoS TCP	0.954	0.963	0.958	46,843
Ransomware	0.941	0.928	0.934	37,468
SQL Injection	0.936	0.942	0.939	31,358
XSS Attack	0.928	0.934	0.931	28,651
Password Attack	0.921	0.918	0.919	25,787
Port Scanning	0.912	0.905	0.908	22,513
Vulnerability Scan	0.908	0.896	0.902	19,686
Backdoor	0.932	0.914	0.923	13,565
Fingerprinting	0.897	0.882	0.889	9136
MITM	0.924	0.908	0.916	6912
Uploading Attack	0.886	0.871	0.878	2504
Macro Average	0.930	0.925	0.927	443,840
Weighted Average	0.947	0.947	0.947	443,840

Note: All metrics represent mean values across n = 5 independent runs with different random seeds [42, 123, 456, 789, 1024]. Statistical significance was assessed via paired *t*-tests (α = 0.05, Bonferroni-corrected) on identical test set instances.

**Table 9:** Energy consumption comparison (MJ per 1 KB encryption).

Algorithm	ESP32	Arduino	RPi 4	Jetson	Avg. Reduction	95% CI/*p*-Value
AES-128	2.84	8.92	0.42	0.18	Baseline	
AES-256	3.67	11.24	0.58	0.24	−23.4%	*p* < 0.01
ASCON-128	1.92	5.47	0.31	0.13	+38.6%	*p* < 0.01
PRESENT-80	1.68	4.82	0.28	0.11	+45.2%	*p* < 0.001
SPECK-128	1.54	4.38	0.25	0.10	+49.8%	*p* < 0.001
EECPF-Adaptive	1.49	4.21	0.24	0.09	+52.6%	*p* < 0.001

Note: Values represent mean ± std across n = 5 runs. Statistical significance was determined via paired *t*-tests with Bonferroni correction.

**Table 10:** Latency comparison (ms).

Operation	AES-128	ASCON	EECPF	Improvement	*p*-Value
Key Generation	12.4	8.7	9.2	25.8%	*p* < 0.01
Encryption (1 KB)	3.8	2.4	2.1	44.7%	*p* < 0.001
Decryption (1 KB)	3.6	2.3	2.0	44.4%	*p* < 0.001
Authentication	45.2	38.6	31.4	30.5%	*p* < 0.001
FL Inference			4.8		
Total Pipeline	65.0	52.0	49.5	23.8%	*p* < 0.001

Note: Values represent mean ± std across n = 5 runs with seeds [42, 123, 456, 789, 1024]. *p*-values were obtained from paired *t*-tests comparing EECPF vs each baseline.

**Table 11:** AESM overhead sensitivity to message frequency.

Msg Rate (msg/s)	Portfolio: 3 Alg (Time%/Energy%/Net%)	Portfolio: 5 Alg (Time%/Energy%/Net%)	Portfolio: 7 Alg (Default) (Time%/Energy%/Net%)	Portfolio: 9 Alg (Time%/Energy%/Net%)	Portfolio: 12 Alg (Time%/Energy%/Net%)
0.1	0.04/0.02/47.3	0.05/0.03/47.2	0.06/0.04/47.1	0.07/0.05/47.0	0.09/0.06/46.9
10	0.51/0.31/47.0	0.58/0.35/46.9	0.63/0.38/46.8	0.71/0.42/46.6	0.84/0.49/46.4
100	1.12/0.76/46.3	1.26/0.85/46.1	1.35/0.91/45.9	1.48/1.01/45.6	1.67/1.14/45.2
1000	9.8/6.2/41.8	10.9/6.9/40.4	11.8/7.4/39.2	13.1/8.2/37.8	15.2/9.6/35.1
10,000	82.4/60.1/−18.2	91.3/67.8/−22.1	98.6/72.3/−25.1	108.7/79.4/−29.3	124.5/88.7/−35.8

Note: Columns show overhead time (%)/overhead energy (%)/net energy saving (%). Portfolio size indicates the number of cryptographic algorithms in AESM’s selection pool. Default configuration uses seven algorithms to balance diversity and computational efficiency.

**Table 12:** Comprehensive comparison with baseline methods.

Method	Year	Accuracy	F1-Score	AUC	Energy (mJ)	Latency (ms)	PQ-Secure	Privacy	Adaptive	*p*-Value ^†^
Haque et al. [2]	2024	89.2%	0.884	0.923	3.42	78.4	No	No	No	<0.001
Zaheer et al. [4]	2024	87.6%	0.869	0.912	2.87	82.1	No	No	No	<0.001
Radhakrishnan et al. [8]	2024	91.3%	0.908	0.948	2.12	56.3	No	No	Partial	<0.01
Dahiphale et al. [9]	2025	88.4%	0.876	0.921	1.89	48.7	No	No	No	<0.01
Popoola et al. [12]	2024	90.8%	0.901	0.942	2.34	61.2	No	Partial	No	<0.01
Ma et al. [17]	2024	86.9%	0.862	0.908	2.56	72.8	Yes	No	No	<0.001
Albogami. [27]	2025	91.3%	0.907	0.951	3.18	68.4	No	Yes	No	<0.01
Mothukuri et al. [29]	2022	89.7%	0.891	0.934	3.45	74.2	No	Yes	No	<0.001
Salim et al. [31]	2024	88.2%	0.874	0.918	2.28	58.9	No	Partial	No	<0.01
Mishra et al. [34]	2025	90.1%	0.894	0.938	2.67	64.3	Partial	No	No	<0.01
EECPF (Ours)	2025	94.7%	0.943	0.987	1.49	49.5	Yes	Yes	Yes	<0.001

^†^ Statistical significance was obtained from paired *t*-tests across n = 5 independent runs (seeds: 42, 123, 456, 789, 1024) on identical dataset splits. All metrics are reported as mean ± std where applicable.

**Table 13:** Ablation study results.

Variant	Accuracy (%)	Energy (mJ)	Latency (ms)	Quantum-Secure	Privacy
EECPF-Full	94.7 ± 0.3	8.4 ± 0.7	23.6 ± 2.1	Yes	Yes
EECPF-Static	94.2 ± 0.4	15.9 ± 0.4	31.0 ± 1.8	Yes	Yes
EECPF-NoFL	95.1 ± 0.2	8.1 ± 0.6	22.3 ± 1.9	Yes	No
EECPF-Classical	94.6 ± 0.3	7.2 ± 0.5	19.8 ± 1.6	No	Yes
EECPF-NoProx	92.8 ± 0.8	8.5 ± 0.7	23.9 ± 2.2	Yes	Yes
EECPF-UniformAgg	93.5 ± 0.6	8.7 ± 0.8	24.1 ± 2.3	Yes	Yes
EECPF-FixedWeights	94.4 ± 0.4	11.6 ± 0.9	26.8 ± 2.4	Yes	Yes
EECPF-RuleBased	94.3 ± 0.4	10.2 ± 0.8	25.4 ± 2.2	Yes	Yes

Note: This ablation study expands upon the preliminary analysis in earlier drafts and now includes seven variants to comprehensively evaluate each component’s contribution to overall performance. All configurations evaluated with n = 5 runs. *p*-values were obtained from paired *t*-tests comparing each ablation against the full EECPF model. EECPF-RuleBased replaces the multi-objective optimization solver with a rule-based selection policy using threshold-based decision trees: if θ > 0.7, then AES-256; else if B_a_ < 0.2, then ASCON-128; otherwise, ChaCha20. This variant tests AESM performance without neural network-based optimization while maintaining adaptive behavior.

**Table 14:** Cross-dataset performance (mean ± std, N = 5 runs).

Dataset	Accuracy	Precision	Recall	F1-Score	AUC-ROC	Energy (mJ)	Latency (ms)
Edge-IIoTset	94.7 ± 0.3	93.8 ± 0.4	94.2 ± 0.3	94.0 ± 0.3	0.982	8.4 ± 0.7	23.6 ± 2.1
CICIDS2017	92.3 ± 0.5	91.5 ± 0.6	91.9 ± 0.5	91.7 ± 0.5	0.968	8.9 ± 0.8	24.2 ± 2.3
UNSW-NB15	89.8 ± 0.7	88.6 ± 0.8	89.1 ± 0.7	88.9 ± 0.7	0.954	9.2 ± 0.9	25.1 ± 2.5

## Data Availability

The dataset used in this study are publicly Available online: https://www.kaggle.com/datasets/mohamedamineferrag/edgeiiotset-cyber-security-dataset-of-iot-iiot (accessed on 20 October 2025).

## Nomenclature

**Symbol**	**Description**
C	Set of available cryptographic algorithms
$c_{i}$	Individual cryptographic algorithm
$E_{i}\left(\cdot \right)$	Energy consumption function for algorithm i
$S_{i}$	Security strength of algorithm i
$L_{i}\left(\cdot \right)$	Latency function for algorithm i
$r_{d}$	Device capability vector
$\left|m\right|$	Message length in bytes
$f\left(r_{d}\right)$	Device capability factor
$K_{i}$	Key setup cost for algorithm i
$p_{d}$	Processing speed of device d
$M_{d}$	Available memory of device d
$B_{d}$	Current battery level of device d
$k_{i}$	Key length of algorithm i
$\tau_{i}$	Algorithm maturity factor
$\nu_{i}$	Known vulnerability score
$\phi_{i}^{\left(q\right)}$	Quantum resistance factor
θ	Normalized threat level (scalar, AESM)
θ	Neural network model parameters (vector, FLIDM)
$L\left(\cdot \right)$	Loss function
$\eta_{t}$	Learning rate at iteration t
$\omega_{k}$	Contribution weight for client k
$q_{k}$	Data quality score for client k
μ	Proximal regularization coefficient
A	LWE public matrix
s	LWE secret vector
e	LWE error vector
q	LWE modulus
χ	Discrete Gaussian distribution
δ_i_	Ciphertext expansion for algorithm i (bytes)
ζ	Latency sensitivity indicator
κ	Threat-security scaling coefficient
λ_1_	Memory penalty coefficient in device capability factor
λ_2_	Battery penalty coefficient in device capability factor
μ	Battery decay factor (Equation (2)); proximal regularization coefficient (Equation (19))
ω_i_	Base encryption time coefficient for algorithm i
ρ_i_	Complexity exponent for algorithm i (power–law)
ψ^E^	Energy priority weight in AESM
ψ^s^	Security priority weight in AESM
ψ^L^	Latency priority weight in AESM
σ_h_	High-value asset sensitivity factor
Sbase	Baseline minimum security requirement
R(n)	Network transmission rate as function of network state
Acronym	Full Form
EECPF	Energy-Efficient Cryptographic Protocol Framework
AESM	Adaptive Encryption Selection Module
FLIDM	Federated Learning Intrusion Detection Module
PQAM	Post-Quantum Authentication Module
EMM	Energy Management Module
IoT	Internet of Things
IIoT	Industrial Internet of Things
LWE	Learning with Errors
KEM	Key Encapsulation Mechanism
DPoS	Delegated Proof-of-Stake
AES	Advanced Encryption Standard
ECC	Elliptic Curve Cryptography
FL	Federated Learning
DDoS	Distributed Denial of Service
ROC	Receiver Operating Characteristic
AUC	Area Under Curve

## Appendix A. Algorithmic Details

**Algorithm A1:** EECPF Main Protocol
**Input:** Device state rd Network state n Message m **Output:** Encrypted message c Authentication token τ
1. Initialize FLIDM with global model parameters θ
2. Collect network traffic features x
3. θ← FLIDM.predict_threat(x, θ)
4. Compute security requirement $S_{min}\left(\theta \right)$ via (10)
5. Compute energy budget $E_{\mathrm{budget}}\left(B_{d}\right)$ 6. for each algorithm ci ∈ C do
7. Compute $E_{i}\left(m,r_{d}\right)$ via Equation (1)
8. Compute $L_{i}\left(m,r_{d},n\right)$ via Equation (4)
9. Compute objective $J\left(c_{i}\right)$ via Equation (11)
10. end for 11. c* ← arg min{ci} J(ci) subject to Constraints (7)–(9)
12. c ← Encrypt(m, c*)
13. τ ← PQAM.authenticate$\left(ID_{d},pk_{d}\right)$
14. Return c, τ

**Algorithm A2:** Federated Learning Training for FLIDM
Local datasets $\{D_{k}{\}}_{k=1}^{K}$,
Initial parameters $\theta^{\left(0\right)}$
Trained global model parameters $\theta^{*}$
Server broadcasts $\theta^{\left(t\right)}$ to all clients $\theta_{k}^{\left(t\right)}\leftarrow \theta^{\left(t\right)}$
$\theta_{k}^{\left(t\right)}\leftarrow \theta_{k}^{\left(t\right)}-\eta_{t}\nabla_{\theta }L_{k}^{\mathrm{prox}}\left(\theta_{k}^{\left(t\right)};B\right)$
Client k sends $\theta_{k}^{\left(t+1\right)}$ to server
Compute quality scores $\{q_{k}{\}}_{k=1}^{K}$
Compute weights $\{\omega_{k}{\}}_{k=1}^{K}$ via [eq:contrib_weight]
$\theta^{\left(t+1\right)}\leftarrow \sum_{k=1}^{K}\omega_{k}\theta_{k}^{\left(t+1\right)}$
End if
End for
$\theta^{\left(T\right)}$

## Appendix B. Reproducibility Specifications

### Appendix B.1. Hardware Configuration

Edge devices included the Raspberry Pi 4 Model B (Broadcom BCM2711, quad-core cortex-A72 @ 1.5 GHz, 4 GB RAM), ESP32-DevKitC (Xtensa LX6 @ 240 MHz, 520 KB SRAM), and Arduino Mega 2560 (ATmega2560 @ 16 MHz, 8 KB SRAM). Server specifications: Intel Xeon E5-2680 v4 @ 2.4 GHz (14 cores), 128 GB DDR4 RAM, NVIDIA Jetson Nano GPU. Energy measurements used INA219 current sensors (±3.2 A range, 12-bit ADC, 1 kHz sampling).

### Appendix B.2. Software Environment

Operating systems: Raspbian GNU/Linux 11 (kernel 5.15.84) for the Raspberry Pi, Ubuntu 20.04.5 LTS (JetPack 4.6.1) for the Jetson Nano, and Ubuntu 22.04.1 LTS (kernel 5.15.0-58) for the server. Python 3.10.12 was used with numpy 1.24.3, torch 2.0.1+cu118, scikit-learn 1.3.0, flwr 1.5.0, and pycryptodome 3.19.1. Cryptographic libraries: OpenSSL 3.0.2 and liboqs 0.9.0. Compilers: GCC 11.4.0 (-O3 -march = native) and NVCC 11.8.89.

### Appendix B.3. Random Seed Configuration

Deterministic seeding ensured reproducibility: random.seed(42), np.random.seed(42), torch.manual_seed(42), and torch.backends.cudnn.deterministic = True. Multi-run experiments (n = 5) used seeds [42, 123, 456, 789, 1024].

### Appendix B.4. Neural Network Architecture

FLIDM employed a four-layer feedforward network: an input layer (61 dimensions), hidden layers (256, 128, and 64 neurons with ReLU, batch normalization, and dropouts of 0.3/0.2/0.1), and an output layer (15 classes, softmax). He initialization was applied. Adam optimizer (β_1_ = 0.9, β_2_ = 0.999, ε = 1 × 10^−8^, weight decay 1 × 10^−5^) was applied. Cosine annealing learning rate schedule (η_max_ = 0.001, η_min_ = 0.0001, T = 100 rounds) was applied. Cross-entropy loss with label smoothing (ε = 0.1) was applied.

### Appendix B.5. Federated Learning Configuration

Non-IID data partitioning was performed via a Dirichlet distribution (α = 0.5) across K = 10 clients. Training parameters: T = 100 communication rounds, E_local = five local epochs, batch size 64, FedProx proximal term μ = 0.01. Gradient compression: top 10% sparsification with 8-bit quantization. Convergence criterion: validation loss change < 0.001 for 10 consecutive rounds.

### Appendix B.6. Measurement Protocols

Energy was measured using the INA219 configured for RANGE_16V, GAIN_8_320MV, and ADC_12BIT. Procedure: record idle power, execute operation, compute energy as (avg_power − idle_power) × duration, and average over 1000 trials. Latency was measured using time.perf_counter() in milliseconds and averaged over 1000 trials.

### Appendix B.7. Complete Parameter Summary

AESM: seven algorithms with weights ψ^E^ = 0.4/ψ^s^ = 0.4/ψ^L^ = 0.2, with S_base = 0.7, and Δθ = 0.15. PQAM: n = 512, q = 12,289, σ = 3.2, matrix 640 × 640. EMM: 15% energy reserve and a 5 s monitoring interval. All parameter values are listed to ensure exact experimental replication.

### Appendix B.8. Code Availability

**Complete implementation:** https://github.com/abdullahtsu/EECPF (accessed on 12 December 2025).
